# Content Variations in Oleocanthalic Acid and Other Phenolic Compounds in Extra-Virgin Olive Oil during Storage

**DOI:** 10.3390/foods11091354

**Published:** 2022-05-06

**Authors:** Jasmine Esposito Salsano, Maria Digiacomo, Doretta Cuffaro, Simone Bertini, Marco Macchia

**Affiliations:** 1 Department of Pharmacy, University of Pisa, Via Bonanno 6, 56126 Pisa, Italy; ja.espositosalsano@student.unisi.it (J.E.S.); doretta.cuffaro@unipi.it (D.C.); simone.bertini@unipi.it (S.B.); marco.macchia@unipi.it (M.M.); 2 Doctoral School in Life Sciences, University of Siena, Via A. Moro 2, 53100 Siena, Italy; 3 Interdepartmental Research Center “Nutraceuticals and Food for Health”, University of Pisa, 56100 Pisa, Italy

**Keywords:** phenolic compound, oleocanthalic acid, oxidative process, hydrolytic process, EVOO storage

## Abstract

The health benefits of extra-virgin olive oil (EVOO) are strictly linked to the presence of phenolic compounds, which exhibit numerous nutraceutical properties. In EVOO, the most important class of phenolic compounds is represented by secoiridoids (oleacein and oleocanthal). EVOO is constantly subjected to degradation processes, including hydrolytic and oxidative reactions that influence its phenolic composition. In particular, the hydrolytic reactions determine the transformation of oleocanthal and oleacein into the corresponding phenyl-alcohols, tyrosol, and hydroxytyrosol. Furthermore, oleocanthal by oxidation processes can be converted to oleocanthalic acid. In this study, we evaluated the phenolic composition of three EVOO samples kept at different storage conditions for 15 months, focusing on the variation of oleocanthalic acid content. Specifically, the samples were stored at 4 °C in darkness and at 25 °C with light exposure. The results of our analyses highlighted that in EVOOs exposed to light and maintained at 25 °C, the degradation was more marked than in EVOO stored in dark and at 4 °C, due to the greater influence of external factors on storage conditions. Although chemical–physical characteristics of EVOOs are slightly different depending on provenience and treatment time, the results of this study reveal that storage conditions are fundamental to controlling phenol concentration.

## 1. Introduction

The beneficial effects of the Mediterranean diet in terms of longevity and reduction in morbidity and mortality are extensively documented, and they are correlated to the use of extra-virgin olive oil (EVOO) as the main source of fats. In the chemical composition of EVOO, a saponifiable (about 98–99%) and an unsaponifiable fraction (about 1–2%) can be distinguished. The saponifiable fraction consists of triglycerides in form of saturated fatty acids (~20%), such as stearic and palmitic acids, unsaturated fatty acids (~70%), among which the most representative is oleic acid and polyunsaturated fatty acids (~10%), such as linoleic and linolenic acids. The unsaponifiable fraction includes more than 230 chemical compounds belonging to different classes such as aliphatic and triterpenic alcohols, phytosterols, pigments, vitamins, and phenolic compounds. The phenolic compounds include secoiridoids, lignans, phenyl-alcohols, phenyl-acids, and flavonoids. Secoiridoids are the most important class of phenolic compounds in EVOO, and they are present exclusively in plants belonging to the *Oleaceae* family. The main secoiridoids present in fresh EVOOs are the dialdehydic form of decarboxymethyl oleuropein aglycone (oleacein), and the dialdehydic form of decarboxymethyl ligstroside aglycone (oleocanthal), which are derived from enzymatic degradation of the glycosides forms of oleuropein and ligstroside, respectively. Lignans represent, after secoiridoids, an abundant class of phenolic compounds in EVOO, and the most representative are pinoresinol and 1-acetoxypinoresinol. Moreover, in EVOO, it is possible to identify flavonoids (e.g., luteolin and apigenin), phenyl-acids (e.g., caffeic, ferulic, *p*-coumaric, and vanillic acids), and phenyl-alcohols, such as hydroxytyrosol and tyrosol.

It has been observed that the health properties of EVOO are strictly related to the presence of phenolic compounds [1]. Indeed, they display numerous nutraceutical activities, including anti-inflammatory, hypotensive, cardioprotective, neuroprotective, antiproliferative, and proapoptotic effects [2,3,4]. Recently, the European Food Safety Authority (EFSA) has authorized some health claims for phenolic compounds such as “Olive oil polyphenols contribute to the protection of blood lipids from oxidative stress” and “The claim may be used only for olive oil which contains at least 5 mg of hydroxytyrosol and its derivatives (e.g., oleuropein complex and tyrosol) per 20 g of olive oil” [5].

**Figure 1:**
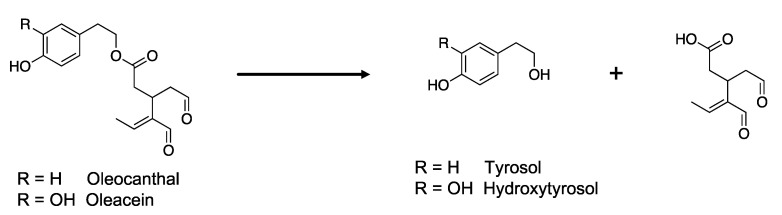
Hydrolytic process that affects oleocanthal and oleacein, leading to the formation of tyrosol and hydroxytyrosol, respectively.

The phenolic content in EVOO is qualitatively and quantitatively influenced by different factors: (1) agronomic, which depends on the *Cultivar*, stage of ripeness of the fruit, climatic conditions, and hydric stress; (2) technological, related to manufacturing processes; (3) conservation of the final product. In particular, EVOO is constantly subjected to degradation processes, especially hydrolytic and oxidative reactions that influence its phenolic composition. The hydrolytic reactions are more closely linked to storage determining the transformation of oleocanthal and oleacein into the corresponding phenyl-alcohols, tyrosol, and hydroxytyrosol (Figure 1).

In fact, several studies highlighted how the concentration of oleocanthal and oleacein is generally higher in fresh EVOOs but decreases during storage as a consequence of hydrolytic processes [6,7]. Moreover, the degradation processes are closely related to the initial phenolic composition [8,9]. Indeed, EVOOs with high initial amounts of phenols, during storage show a lower decrease in phenolic content than EVOOs characterized by low initial amounts of phenolic compounds [8].

External factors such as light, temperature, and oxygen, as well as other pro-oxidant activators (such as chlorophylls), influence EVOO by increasing the kinetics of oxidative reactions. Furthermore, it has been observed that auto-oxidation phenomena occur naturally in EVOO, even under controlled conditions [6,8].

**Figure 2:**
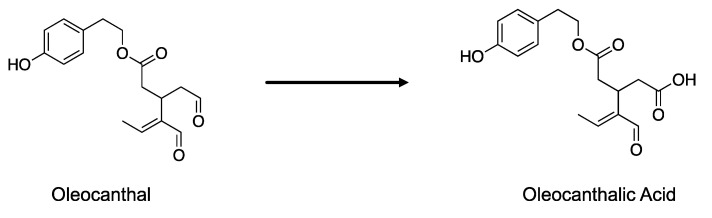
Oxidative process leads to the formation of oleocanthalic acid from oleocanthal.

Recently, the product of the oxidation of oleocanthal—namely, oleocanthalic acid (Figure 2)—was identified in an EVOO stored for 24 months, and its neuroprotective activity has been proved [10].

In our previous paper, we developed a reproducible and efficient procedure to isolate and purify oleocanthalic acid and an HPLC method to analyze the content of oleocanthalic acid in EVOO [11]. The aim of this study was to evaluate the variations in oleocanthalic acid content in some EVOOs produced in different crop seasons and stored at different temperatures over 15 months. With the aim to compare the progression of oxidative and hydrolytic processes during the storage, the amounts of oleocanthal, oleacein, tyrosol, and hydroxytyrosol were also monitored.

## 2. Materials and Methods

### 2.1. Solvents and Analytical Standards

Acetonitrile (ACN), methanol (MeOH), H_2_O, acetic acid (AcOH), and *n*-hexane were purchased from Sigma-Aldrich (Milan, Italy). The following standards were used: hydroxytyrosol and tyrosol were purchased from TCI (Zwijndrecht, Belgium); *p*-hydroxyphenyl acetic acid (internal standard (IS)) and gallic acid were purchased from Sigma Aldrich (Milan, Italy); oleocanthal, oleacein, and oleocanthalic acid were isolated and purified by us, as reported in our previous studies [11,12,13]. Folin–Cicalteu’s phenol reagent (FCR) was purchased from Merck (Darmstadt, Germany).

### 2.2. Instruments

Solvent evaporation was achieved under vacuum by using a rotating evaporator (Strike 300, Stereoglass, Perugia, Italy). The homogenization was achieved by using a vortex mixer (Zx3, Advanced Vortex Mixer, VELP^®^ Scientifica, Usmate Velate, Italy) and a rotary shaker. Centrifugation was performed by using a centrifuge 4225 at 4000 rpm.

HPLC analysis was performed with an HPLC instrument (Thermo Finnigan-Spectra System SCM1000, Thermo Electron Corporation, Waltham, MA, USA) equipped with a Spectra System P2000 (Thermo Electron Corporation, Waltham, MA, USA ), Spectra System UV2000, set to 280 nm, and by using a Phenomenex Gemini reverse-phase C18 column (250 × 4.6 mm, 5 μm particle size; Phenomenex, Castel Maggiore, Italy).

Spectrophotometric analysis was carried out with a spectrophotometer (Shimadzu, Columbia, MD, USA) set at 725 nm.

### 2.3. EVOO Samples, Storage Conditions, and Phytoextracts Preparation

Three EVOOs were selected: two Tuscan EVOOs of the 2019/2020 crop seasons (A and B) and an Italian EVOO of the 2018/2019 crop season (C). EVOO samples were stored under different conditions: an aliquot was maintained at 25 °C and daylight exposed; another aliquot was kept at the temperature of 4 °C, in dark conditions. At the beginning of the analysis (December 2019), EVOOs A and B were fresh, while the EVOO C had already been stored for one year at room temperature in dark conditions. In order to evaluate the variation in the phenolic composition, the three EVOO samples were periodically monitored during 15 months of storage (from December 2019 to March 2021), through HPLC analysis.

The phytoextracts of EVOO were prepared as previously described [14]. Briefly, an EVOO sample (3 g) was mixed with *n*-hexane (12 mL) and ACN (15 mL). The resulting mixture was homogenized by using a vortex mixer for 30 s and a rotary shaker for 30 min. Then, after centrifugation at 4000 rpm for 5 min, the ACN phase was collected and evaporated under reduced pressure to afford the phytoextract.

### 2.4. Analysis of Phenolic Compounds Content

HPLC analysis of EVOO samples was carried out by using a slightly modified method from that developed in our previous study [7,11].

The phytoextracts of EVOO, prepared as described in Section 2.3, were injected in HPLC as a mixture of MeOH/ H_2_O (1:1 *v*/*v*). The mobile phase was a mixture of H_2_O/AcOH (97.5:2.5 *v*/*v*) (A) and ACN/MeOH (1:1 *v*/*v*) (B). A linear gradient was run from 5% (B) to 30% (B) in 45 min; it changed to 70% (B) during 20 min (65 min total time); in 5 min, it changed to 80% (B) (70 min total time); it remained at 80% (B) for 15 min (85 min total time); it changed to 100% (B) in 5 min (90 min total time); after re-equilibration in 5 min (95 min total time) to initial composition, it remained at 5% (B) for 10 min (105 min total time), as shown in Table 1. The flow rate was 1 mL/min, and the injected volume was 50.0 μL.

The retention times and UV absorbance spectra of phenolic compounds were compared with those of standards and quantified at 280 nm, by using *p*-hydroxyphenyl acetic acid as the internal standard. For each analyzed compound, the calibration curve was built, and the limit of detection (LOD) and the limit of quantification (LOQ) were determined [7,11]. For each calibration curve, the correlation coefficients (r^2^) were >0.999 [7].

The phenolic compound concentration was expressed as µg of phenolic compound/g of EVOO (ppm).

### 2.5. Determination of Total Phenolic Content

The TPC was determined with the Folin–Ciocalteu assay, by following a procedure reported in the literature [15], with slight modifications. EVOO sample (2.5 g) was dissolved in 5 mL of *n*-hexane and extracted with 5 mL of MeOH (80% *v*/*v*) by using a vortex mixer for 1 min and a rotary shaker for 15 min. After centrifugation for 5 min at 4000 rpm at 25 °C, the MeOH phase was collected. This procedure was repeated twice, obtaining 10 mL of final methanolic solution. Then, 0.25 mL of FCR and 1.5 mL of Na_2_CO_3_ (20% *w*/*v*) were added to 1 mL of the methanolic solution in a volumetric flask, after which distilled water was added, reaching the final volume of 10 mL. The resulting mixture was kept for 45 min at the controlled temperature of 25 °C, on a stove (Heraeus). Spectrophotometric analysis was performed at λ = 725 nm. Each analysis was executed in triplicate, and the TPC of each EVOO was expressed as µg of gallic acid equivalent (GAE)/g of EVOO (ppm). In the same way, the calibration curve of gallic acid was built (Table 2).

## 3. Results and Discussion

In this study, the variations in phenolic composition in three different EVOO samples during 15 months of storage were evaluated. The phenolic compounds, tyrosol, hydroxytyrosol, oleocanthal, oleacein, and oleocanthalic acid, were identified and quantified by HPLC analysis (Figures S1–S9).

### 3.1. Initial Phenolic Content in EVOOs

**Figure 3:**
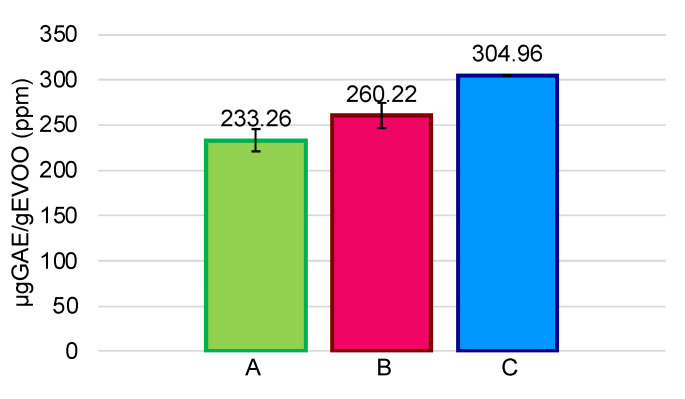
Total phenolic content (TPC) (µg GAE/g EVOO, ppm) in each EVOOs (A, B, and C). Data are expressed as mean ± SD of an experiment performed in triplicate.

The initial TPC ranged from 233.26 µg GAE/g of EVOO for sample A to 304.96 µg GAE /g EVOO for sample C, showing low variability (Figure 3).

However, as regards the number of individual phenols (Table 3 and Table 4), the samples showed some differences, probably related to the olive variety (*Cultivar*) and the aging of the EVOOs, as two (A and B) are Tuscan EVOOs produced in the 2019/2020 crop season, whereas C is an Italian EVOO produced in the 2018/2019 crop season.

Initially, in fresh EVOOs (A and B), oleocanthalic acid was present at a very low concentration (EVOO A, 10.61 ppm), or it was not present (EVOO B), while in EVOO C, which is one year older, already contained oleocanthalic acid (39.72 ppm). These results are in agreement with the data reported in the literature [5]. Regarding oleocanthal and oleacein, EVOOs showed low variability, with a range from 148.93 ppm to 226.36 ppm for oleocanthal and a range from 94.49 ppm to 116.09 ppm for oleacein. A relevant difference concerned tyrosol and hydroxytyrosol content, which was very low in fresh EVOOs A and B, while it was higher in one-year older EVOO C, according to the literature. In fact, several studies showed that fresh EVOO has a high content of oleocanthal and oleacein and a low concentration of tyrosol and hydroxytyrosol because, during storage, the content of oleocanthal and oleacein decreases due to hydrolytic processes, which leads to the formation of tyrosol and hydroxytyrosol, respectively [7].

### 3.2. Variations in the Phenolic Content in EVOOs during Storage

The change in phenolic content in EVOOs during storage for 15 months, at 4 °C in darkness, and at 25 °C exposed to light, was evaluated through HPLC analysis, the results of which are detailed below.

#### 3.2.1. EVOOs Stored at 25 °C and Exposed to Light

**Figure 4:**
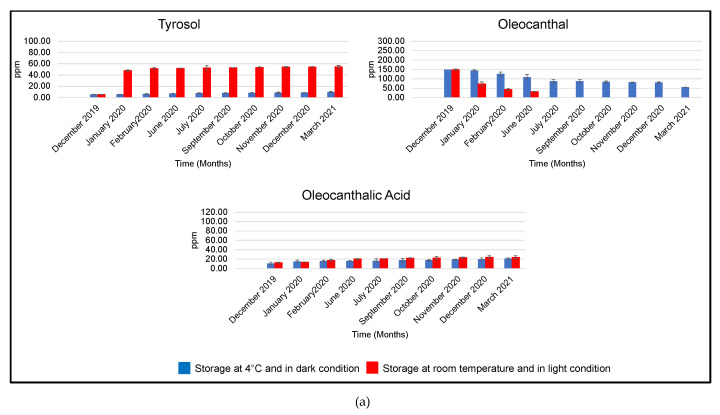

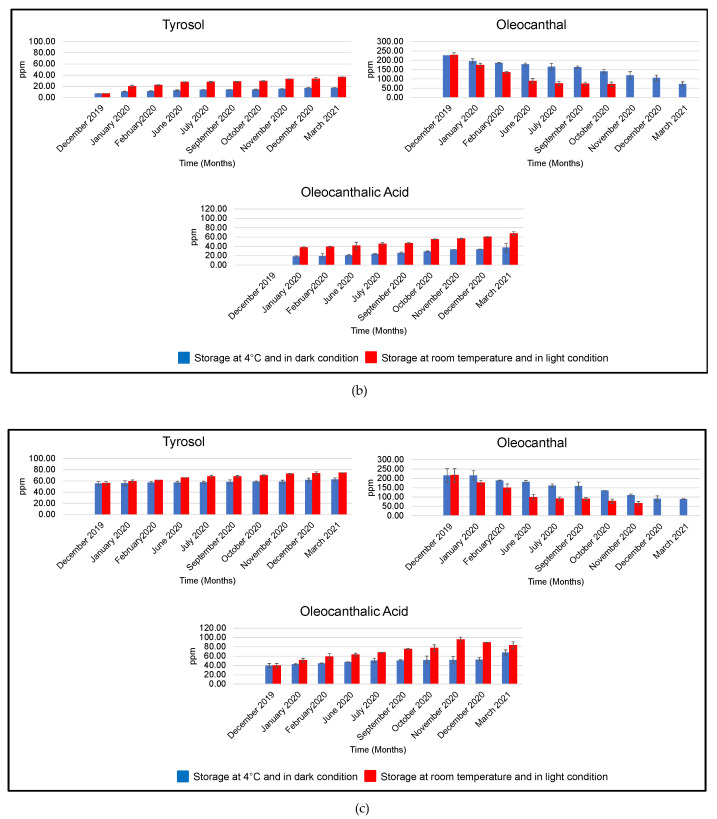
Concentrations of oleocanthal, tyrosol, and oleocanthalic acid (µg of phenolic compound/g of EVOO, ppm) in EVOOs A (**a**), B (**b**), and C (**c**) stored at 4 °C in dark conditions (blue bars) and at room temperature and exposed to daylight (red bars), for 15 months.

In all analyzed EVOOs, oleocanthalic acid content increased during storage at 25 °C under light exposure (Table 4 and Figure 4—red bars, Figure S10).

Interestingly, this trend was marked in sample B, in which, after 15 months of storage, the concentration of oleocanthalic acid increased 7 times, compared with the initial amount. In sample A, the rise was slow, doubling after 6 months, after which it remained unchanged. The cause of this behavior could be the absence of oleocanthal in EVOO after 7 months of storage. In sample C, oleocanthalic acid content doubled after 11 months and then decreased in the following months, probably due to a hydrolytic process that affects oleocanthalic acid itself.

The greater increase in oleocanthalic acid in sample B could be correlated to the higher initial amount of oleocanthal in EVOO.

As regards the variation in oleocanthal and oleacein, during the storage of EVOO at 25 °C under daylight exposure, the concentrations of these compounds decreased in all samples but at different rates (Table 4 and Figure 4). In fact, in EVOO A, oleocanthal and oleacein decreased quickly, and after only 8 months of storage, they were no longer detectable. Conversely, in samples B and C, the two secoiridoids gradually decreased and were undetectable after 11 and 12 months, respectively.

During storage, in parallel with the decrease in the content of oleocanthal and oleacein, the concentrations of simple phenols, tyrosol, and hydroxytyrosol increased, due to hydrolysis of oleocanthal and oleacein in the corresponding phenyl-alcohols. This trend was more evident in EVOO A, in which, after only 2 months of storage, tyrosol and hydroxytyrosol increased, respectively, by 10 and 7 times, compared with their initial amounts, showing a wide hydrolytic process. In EVOO C, the amounts of tyrosol and hydroxytyrosol displayed minimal changes during storage.

In all analyzed EVOOs, tyrosol and hydroxytyrosol concentrations increased until reaching a plateau phase. However, hydroxytyrosol content, after an initial raise, decreased (after 11 months of storage, October 2020). This behavior can be explained by the instability of hydroxytyrosol that can be subjected to degradation processes.

#### 3.2.2. EVOOs Stored at 4 °C and in Darkness

In aliquots stored at 4 °C in darkness, the hydrolytic process was slowed down (Table 3 and Figure 4—blue bars). In all samples, the variations in oleocanthal and oleacein showed a very similar trend, decreasing slowly. The raise of tyrosol and hydroxytyrosol was very gradual, doubling after 15 months in samples A and B and remaining almost constant in sample C.

These results demonstrated that in EVOOs exposed to light and maintained at 25 °C, the degradation, due to a hydrolytic process, was generally more marked than in EVOOs stored in dark and at 4 °C.

Concerning the formation of oleocanthalic acid, in aliquots stored at 4 °C and in the dark, the oxidative process was slower than that at 25 °C, for all samples except for sample A, in which the amount of oleocanthalic acid was comparable with that at 25 °C. Therefore, the oxidative process was not completely influenced by temperature and light. In fact, auto-oxidative processes inside the EVOO may be produced also in the dark.

## 4. Conclusions

In this study, we focused on the variations in the oleocanthalic acid content, a recently identified polyphenol as an oxidation product of oleocanthal, during the storage of three different EVOOs produced in specific crop seasons (2018/2019 and 2019/2020). In parallel, the hydrolytic degradation of oleocanthal and oleacein was investigated. The samples were monitored for 15 months in two different storage conditions: 4 °C in darkness and at 25 °C and exposed to light. The results of HPLC analysis highlighted that EVOO phenols concentration is strictly dependent on the origin and the age of EVOO. Moreover, external factors affecting storage conditions, such as light, temperature, and oxygen, influenced the phenolic content. In EVOOs maintained at room temperature under daylight exposure, variation in the content of phenolic compounds was more marked than that in EVOOs stored in the dark and at 4 °C. In conclusion, in the analyzed EVOOs, the degradation kinetics, and, in particular, hydrolytic kinetics, appeared to be strictly influenced by the storage parameters and by the characteristics of the EVOO (age and composition).

## Figures and Tables

**Table 1:** Mobile phase of HPLC method.

Total Time (min)	A%	B%
0.0	95	5
45.0	70	30
65.0	30	70
70.0	20	80
85.0	20	80
90.0	-	100
95.0	95	5
105.0	95	5

**Table 2:** Calibration curve and r^2^ of gallic acid.

Compound	Calibration Curve	r^2^
Gallic Acid	y = 0.0098 + 0.098	0.995

**Table 3:** Concentrations of phenolic compounds (µg of phenolic compound/g of EVOO, ppm) in three different EVOOs (A, B, and C) storage at 4 °C and in dark conditions. Data are expressed as means ± SD of experiment performed in triplicate.

Month	EVOO	Hydroxytyrosol	Tyrosol	Oleacein	Oleocanthal	Oleocanthalic Acid
December 2019	A	4.72 ± 0.11	5.47 ± 0.25	116.09 ± 18.39	148.93 ± 3.04	10.61 ± 2.96
	B	5.67 ± 0.01	7.28 ± 0.01	110.69 ± 9.80	226.36 ± 13.30	-
	C	34.11 ± 0.57	55.62 ± 3.10	94.49 ± 3.75	216.12 ± 36.44	39.72 ± 4.59
January 2020	A	5.51 ± 0.12	5.86 ± 0.14	103.40 ± 8.20	144.54 ± 10.46	15.20 ± 2.64
	B	7.58 ± 0.29	10.84 ± 0.67	105.01 ± 0.46	194.94 ± 2.73	18.63 ± 1.20
	C	35.78 ± 0.99	55.96 ± 4.36	92.84 ± 3.00	216.00 ± 24.76	42.65 ± 1.07
February 2020	A	5.74 ± 0.18	6.31 ± 0.59	97.67 ± 1.71	125.72 ± 13.67	15.94 ± 1.64
	B	8.41 ± 0.48	11.71 ± 0.75	98.94 ± 5.98	184.92 ± 6.47	19.40 ± 5.22
	C	36.40 ± 0.19	57.04 ± 1.96	90.00 ± 11.43	188.85 ± 1.99	44.42 ± 1.00
June 2020	A	7.40 ± 0.18	7.22 ± 0.16	95.33 ± 2.26	108.82 ± 8.50	16.32 ± 0.28
	B	9.38 ± 0.57	12.76 ± 0.91	91.73 ± 11.47	178.15 ± 17.65	20.76 ± 1.33
	C	36.59 ± 1.31	57.17 ± 2.12	85.67 ± 9.61	182.22 ± 6.60	47.25 ± 0.67
July 2020	A	7.52 ± 0.59	7.72 ± 0.87	71.36 ± 16.28	87.42 ± 9.13	16.44 ± 4.12
	B	10.14 ± 0.50	13.97 ± 0.23	89.15 ± 6.18	165.78 ± 6.77	23.83 ± 0.57
	C	38.73 ± 1.92	57.45 ± 1.89	79.76 ± 8.72	160.95 ± 8.91	50.71 ± 4.62
September 2020	A	7.98 ± 0.12	8.03 ± 0.49	62.31 ± 5.46	86.42 ± 5.36	17.85 ± 3.68
	B	10.77 ± 0.15	14.10 ± 0.22	86.56 ± 4.05	162.87 ± 9.20	26.40 ± 1.81
	C	40.29 ± 2.53	58.30 ± 3.58	76.02 ± 13.20	159.92 ± 20.37	50.89 ± 1.40
October 2020	A	8.68 ± 0.56	8.51 ± 0.23	61.05 ± 7.18	82.93 ± 0.53	17.89 ± 0.76
	B	11.08 ± 0.34	14.46 ± 0.60	75.88 ± 11.64	140.88 ± 19.27	28.90 ± 1.36
	C	33.71 ± 0.80	58.58 ± 1.30	66.60 ± 3.96	135.52 ± 0.06	51.50 ± 8.51
November 2020	A	8.85 ± 0.22	8.59 ± 1.01	49.98 ± 6.12	81.20 ± 3.01	19.09 ± 0.61
	B	11.42 ± 0.57	15.58 ± 0.48	60.02 ± 0.37	119.80 ± 13.71	33.16 ± 0.20
	C	33.50 ± 1.98	58.60 ± 2.77	55.39 ± 5.96	110.86 ± 5.88	52.00 ± 7.34
December 2020	A	8.90 ± 0.04	8.88 ± 0.20	49.98 ± 5.90	80.29 ± 0.40	20.03 ± 2.43
	B	13.18 ± 0.02	17.45 ± 0.80	55.63 ± 9.77	106.64 ± 11.16	33.80 ± 0.25
	C	32.89 ± 0.07	61.73 ± 3.28	51.24 ± 4.67	91.75 ± 15.06	52.68 ± 4.02
March 2021	A	10.10 ± 0.91	9.80 ± 1.09	37.74 ± 4.14	55.34 ± 4.36	21.16 ± 1.42
	B	13.74 ± 0.25	17.52 ± 0.25	39.57 ± 6.39	72.65 ± 0.53	37.39 ± 9.10
	C	31.61 ± 3.04	62.70 ± 2.73	43.66 ± 4.57	89.27 ± 2.60	67.83 ± 5.55

**Table 4:** Concentrations of phenolic compounds (µg of phenolic compound/g of EVOO, ppm) in three different EVOOs (A, B, and C) storage at 25 °C and exposed to light. Data are expressed as means ± SD of experiment performed in triplicate. N.D. = not detectable.

Month	EVOO	Hydroxytyrosol	Tyrosol	Oleacein	Oleocanthal	Oleocanthalic Acid
December 2019	A	4.72 ± 0.11	5.47 ± 0.25	116.09 ± 18.39	148.93 ± 3.04	10.61 ± 2.96
	B	5.67 ± 0.01	7.28 ± 0.01	110.69 ± 9.80	226.36 ± 13.30	-
	C	34.11 ± 0.57	55.62 ± 3.10	94.49 ± 3.75	216.12 ± 36.44	39.72 ± 4.59
January 2020	A	27.30 ± 0.74	47.80 ± 1.21	42.72 ± 3.10	72.45 ± 10.51	13.70 ± 0.17
	B	14.53 ± 0.34	19.73 ± 2.43	83.22 ± 19.83	173.17 ± 11.17	37.54 ± 1.51
	C	36.85 ± 1.70	59.15 ± 2.53	72.24 ± 7.05	176.47 ± 13.33	51.07 ± 4.20
February 2020	A	27.09 ± 0.07	51.66 ± 1.33	40.96 ± 3.99	43.38 ± 5.26	17.48 ± 1.92
	B	16.34 ± 0.18	22.14 ± 0.63	65.30 ± 16.09	134.09 ± 4.94	38.75 ± 1.66
	C	37.06 ± 0.04	61.43 ± 0.02	66.23 ± 12.31	149.64 ± 20.87	58.72 ± 6.79
June 2020	A	29.89 ± 0.05	51.74 ± 0.28	16.20 ± 1.05	31.58 ± 1.15	20.37 ± 0.95
	B	18.93 ± 2.77	27.70 ± 0.61	55.30 ± 19.06	88.56 ± 13.35	41.28 ± 7.31
	C	38.44 ± 3.91	65.76 ± 0.03	52.96 ± 7.33	98.93 ± 15.17	63.13 ± 3.07
July 2020	A	28.64 ± 0.54	52.63 ± 4.18	N.D.	N.D.	20.64 ± 0.55
	B	22.20 ± 4.19	27.78 ± 1.00	43.45 ± 11.00	74.50 ± 11.13	45.26 ± 2.39
	C	40.29 ± 0.35	67.69 ± 2.35	36.93 ± 2.29	91.33 ± 9.36	67.71 ± 0.46
September 2020	A	27.36 ± 1.34	53.14 ± 0.13	N.D.	N.D.	22.04 ± 0.67
	B	21.28 ± 0.17	28.43 ± 0.57	43.37 ± 18.58	73.59 ± 8.80	46.70 ± 1.22
	C	41.78 ± 0.73	67.89 ± 1.89	33.72 ± 19.08	89.72 ± 7.23	75.11 ± 1.23
October 2020	A	27.36 ± 0.42	53.66 ± 1.00	N.D.	N.D.	22.39 ± 3.43
	B	20.86 ± 0.47	29.29 ± 0.71	35.62 ± 10.56	71.31 ± 11.69	54.95 ± 0.83
	C	34.06 ± 0.88	69.77 ± 0.96	33.44 ± 0.40	77.73 ± 11.28	77.16 ± 7.27
November 2020	A	25.30 ± 0.01	54.18 ± 0.91	N.D.	N.D.	23.11 ± 1.25
	B	20.36 ± 0.90	33.09 ± 0.34	N.D.	N.D.	56.64 ± 0.92
	C	31.95 ± 6.21	72.39 ± 0.90	N.D.	66.34 ± 10.36	95.56 ± 5.21
December 2020	A	19.22 ± 0.92	54.51 ± 0.34	N.D.	N.D.	23.96 ± 2.98
	B	20.18 ± 0.63	33.54 ± 2.72	N.D.	N.D.	60.39 ± 0.68
	C	31.88 ± 7.04	73.47 ± 2.78	N.D.	N.D.	89.11 ± 0.15
March 2021	A	17.91 ± 2.80	54.76 ± 2.25	N.D.	N.D.	24.00 ± 6.85
	B	18.50 ± 0.29	36.63 ± 0.19	N.D.	N.D.	67.23 ± 3.84
	C	30.49 ± 1.24	74.38 ± 0.06	N.D.	N.D.	83.27 ± 7.56

## Data Availability

Data is contained within the article or Supplementary Material.

## Supplementary Materials

The following supporting information can be downloaded at: https://www.mdpi.com/article/10.3390/foods11091354/s1, Figure S1: HPLC chromatogram of EVOO A at the beginning of the analysis (December 2019). IS = Internal Standard; Figure S2: HPLC chromatogram of EVOO B at the beginning of the analysis (December 2019). IS = Internal Standard; Figure S3: HPLC chromatogram of EVOO C at the beginning of the analysis (December 2019). IS = Internal Standard; Figure S4: HPLC chromatogram of EVOO A, analysed after fifteen months of storage at room temperature and in light condition (March 2021). IS = Internal Standard; Figure S5: HPLC chromatogram of EVOO B, analysed after fifteen months of storage at room temperature and in light condition (March 2021). IS = Internal Standard; Figure S6: HPLC chromatogram of EVOO C, analysed after fifteen months of storage at room temperature and in light condition (March 2021). IS = Internal Standard; Figure S7: HPLC chromatogram of EVOO A analysed after fifteen months of storage at 4 °C and in dark condition (March 2021). IS = Internal Standard; Figure S8: HPLC chromatogram of EVOO B analysed after fifteen months of storage at 4 °C and in dark condition (March 2021). IS = Internal Standard; Figure S9: HPLC chromatogram of EVOO C analysed after fifteen months of storage at 4 °C and in dark condition (March 2021). IS = Internal Standard; Figure S10: Concentration of hydroxytyrosol and oleacein (μg of phenolic compound/g of EVOO, ppm) in EVOOs A (a), B (b) and C (c) stored at 4 °C in dark condition (blue bars) and at room temperature exposed to daylight (red bars), for 15 months.
