# Supplementary material for: Content Variations in Oleocanthalic Acid and Other Phenolic Compounds in Extra-Virgin Olive Oil during Storage
Source: Foods. 2022 May 6;11(9):1354. doi: 10.3390/foods11091354 (PMC9105779; doi:10.3390/foods11091354)
Supplement: Supplementary file 1 [file foods-11-01354-s001.zip › Figure S10 (a).pdf]

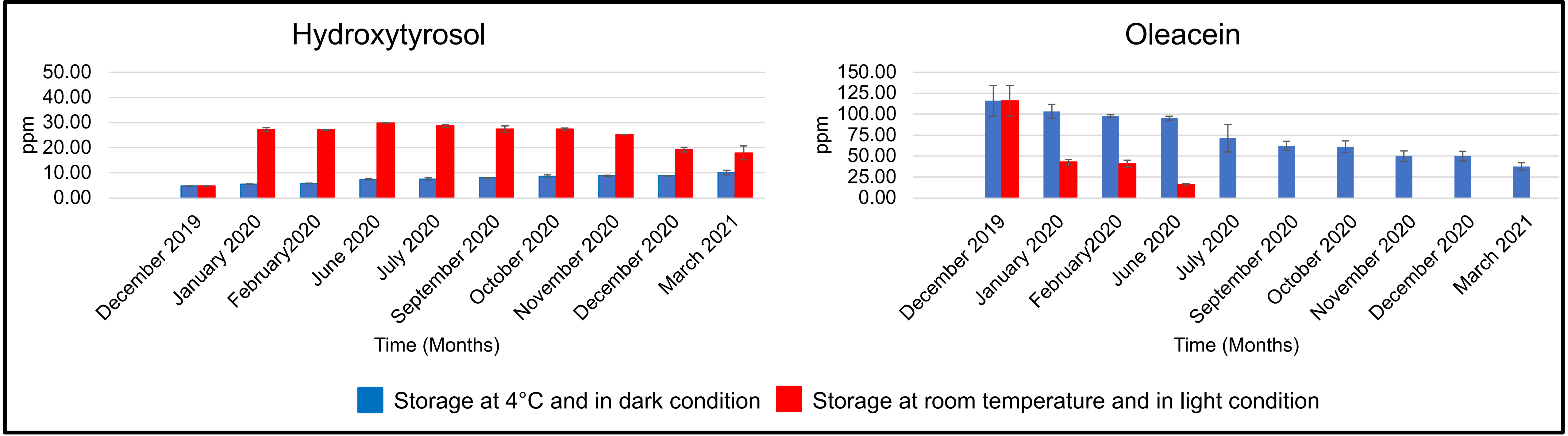

Hydroxytyrosol
50.00
40.00
30.00
20.00
10.00
0.00
ppm
December 2019
January 2020
February2020
June 2020
July 2020
September 2020
October 2020
November 2020
December 2020
March 2021
Time (Months)
Oleacein
150.00
125.00
100.00
75.00
50.00
25.00
0.00
ppm
December 2019
January 2020
February2020
June 2020
July 2020
September 2020
October 2020
November 2020
December 2020
March 2021
Time (Months)
Storage at 4°C and in dark condition
Storage at room temperature and in light condition

(a)
