# Supplementary figures and images for: Content Variations in Oleocanthalic Acid and Other Phenolic Compounds in Extra-Virgin Olive Oil during Storage
Source: Foods. 2022 May 6;11(9):1354. doi: 10.3390/foods11091354 (PMC9105779; doi:10.3390/foods11091354)

# EVOO A

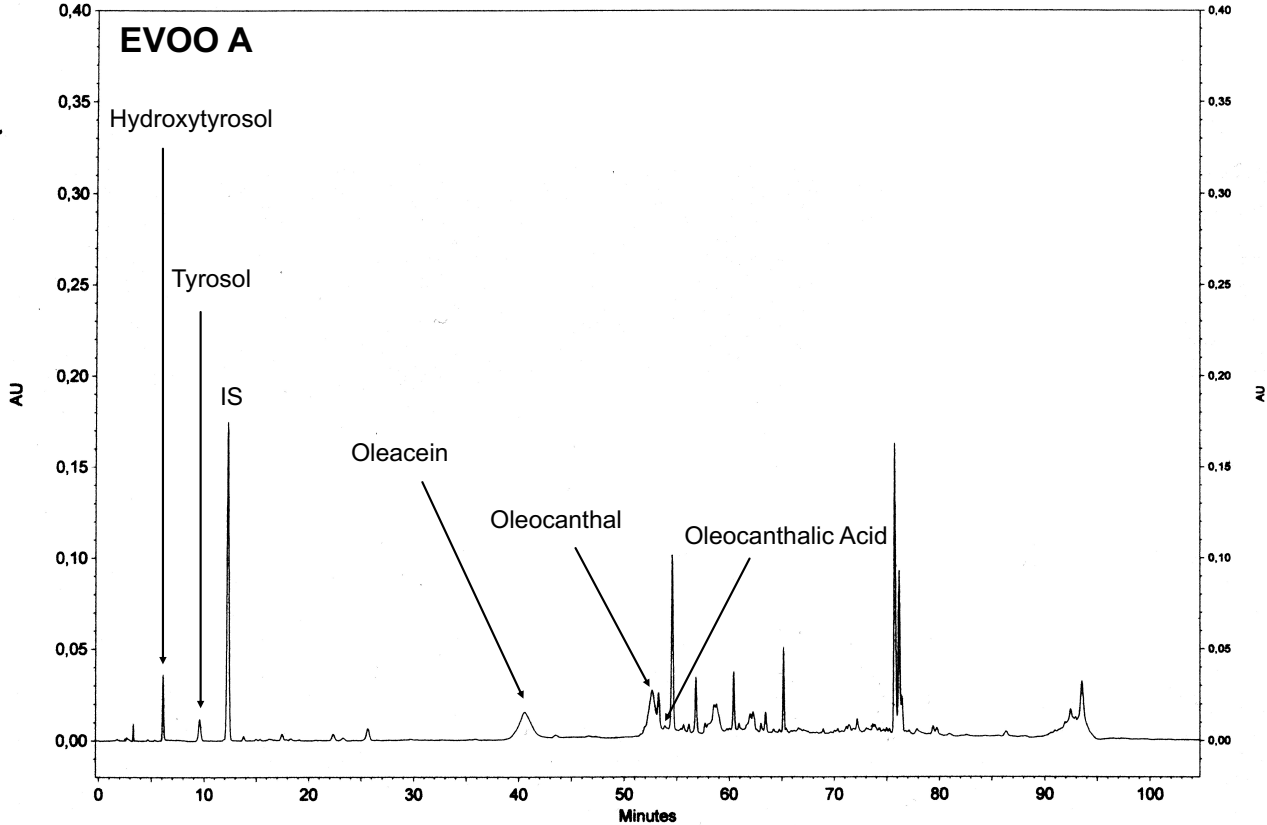

Supplement: Supplementary file 1 [file foods-11-01354-s001.zip › Figure S1.pdf]

# EVOO B

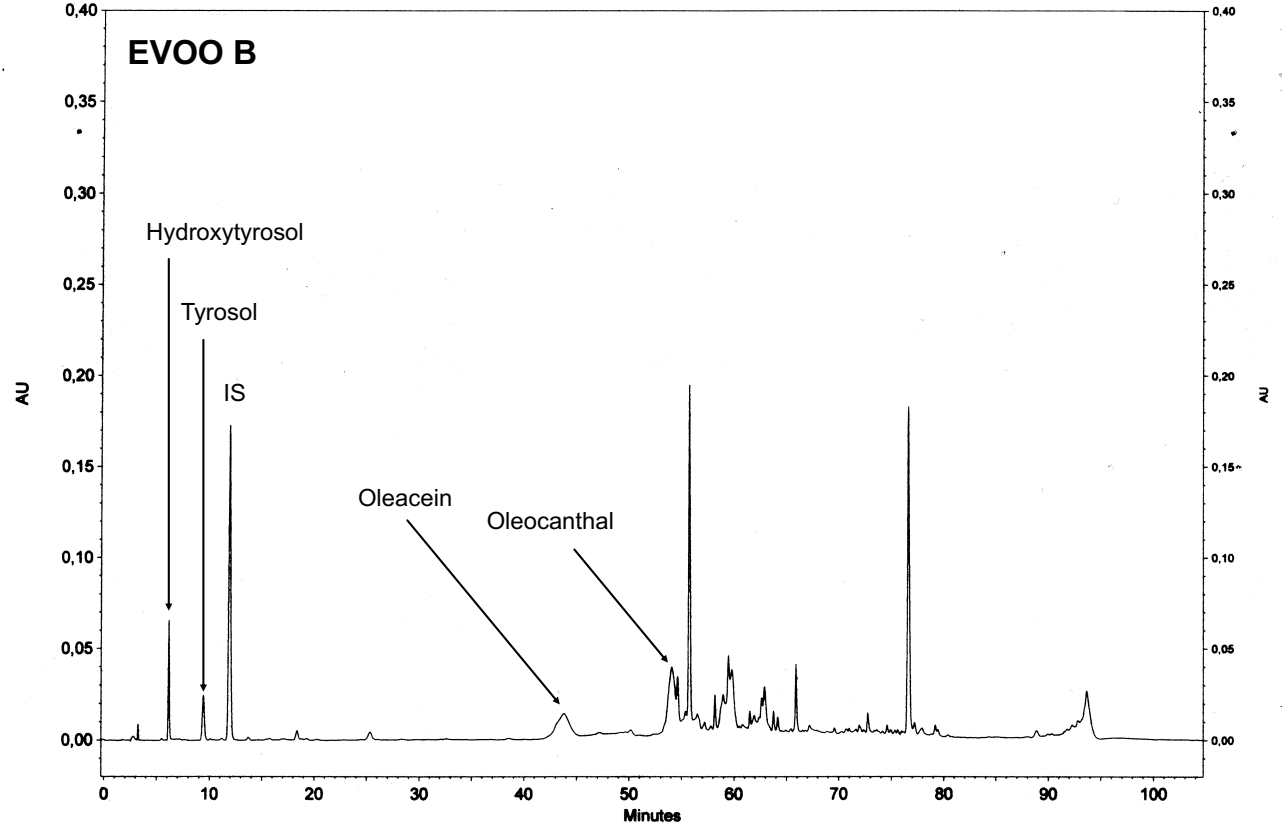

Supplement: Supplementary file 1 [file foods-11-01354-s001.zip › Figure S2.pdf]

# EVOO C

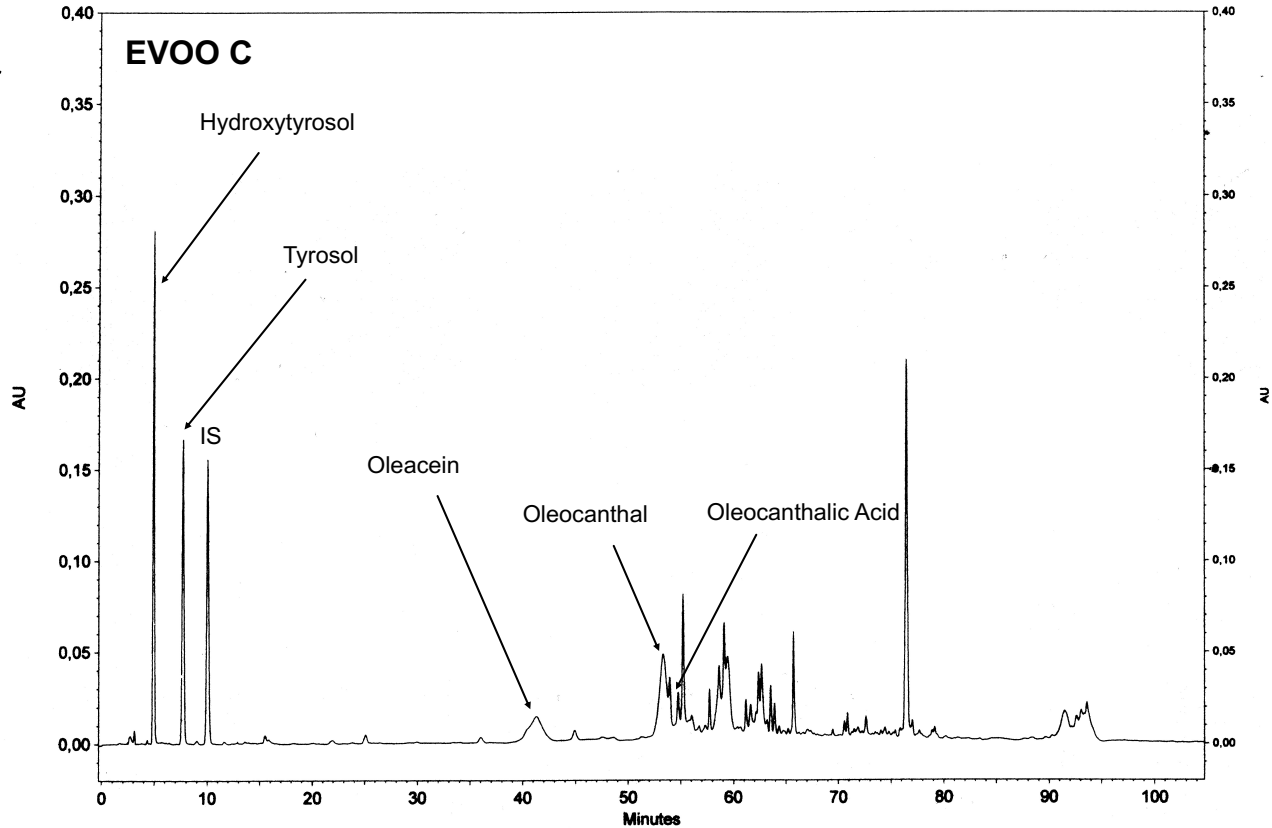

Supplement: Supplementary file 1 [file foods-11-01354-s001.zip › Figure S3.pdf]

# EVOO A

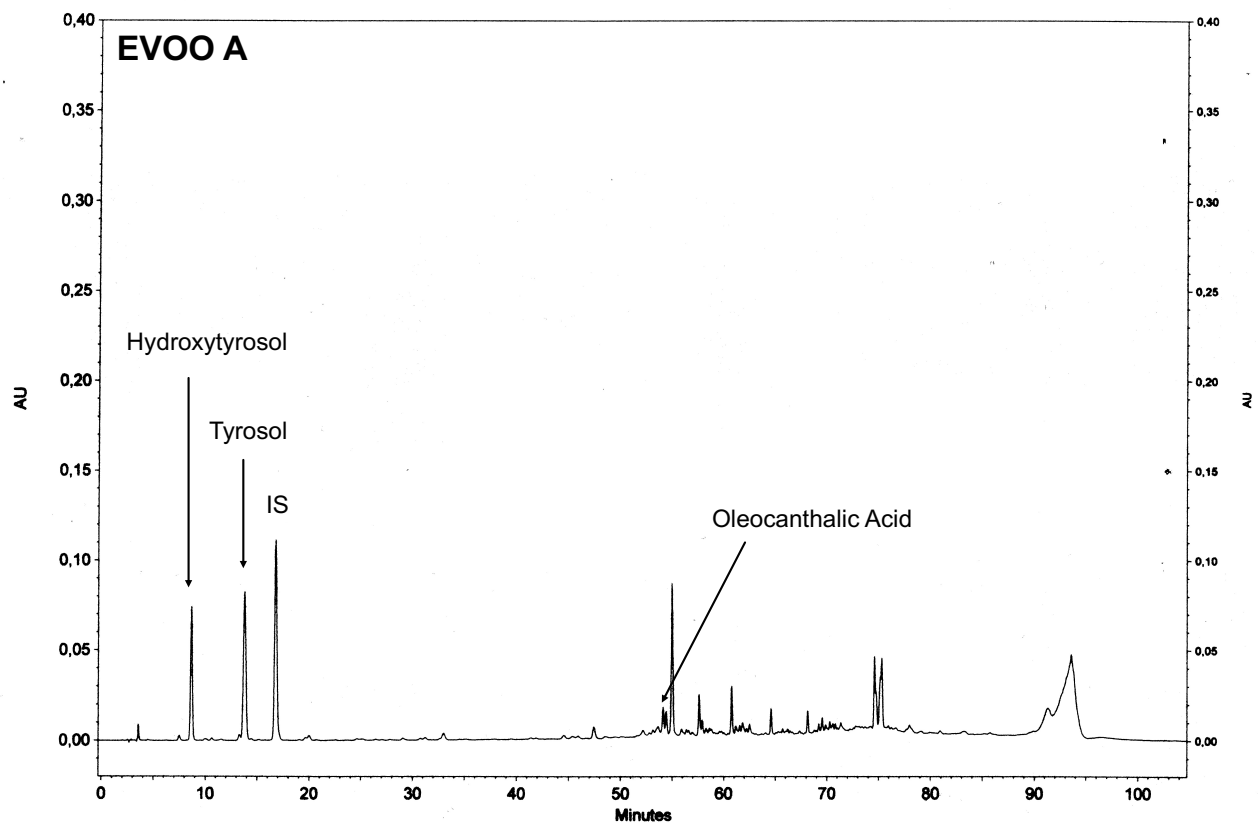

Supplement: Supplementary file 1 [file foods-11-01354-s001.zip › Figure S4.pdf]

# EVOO B

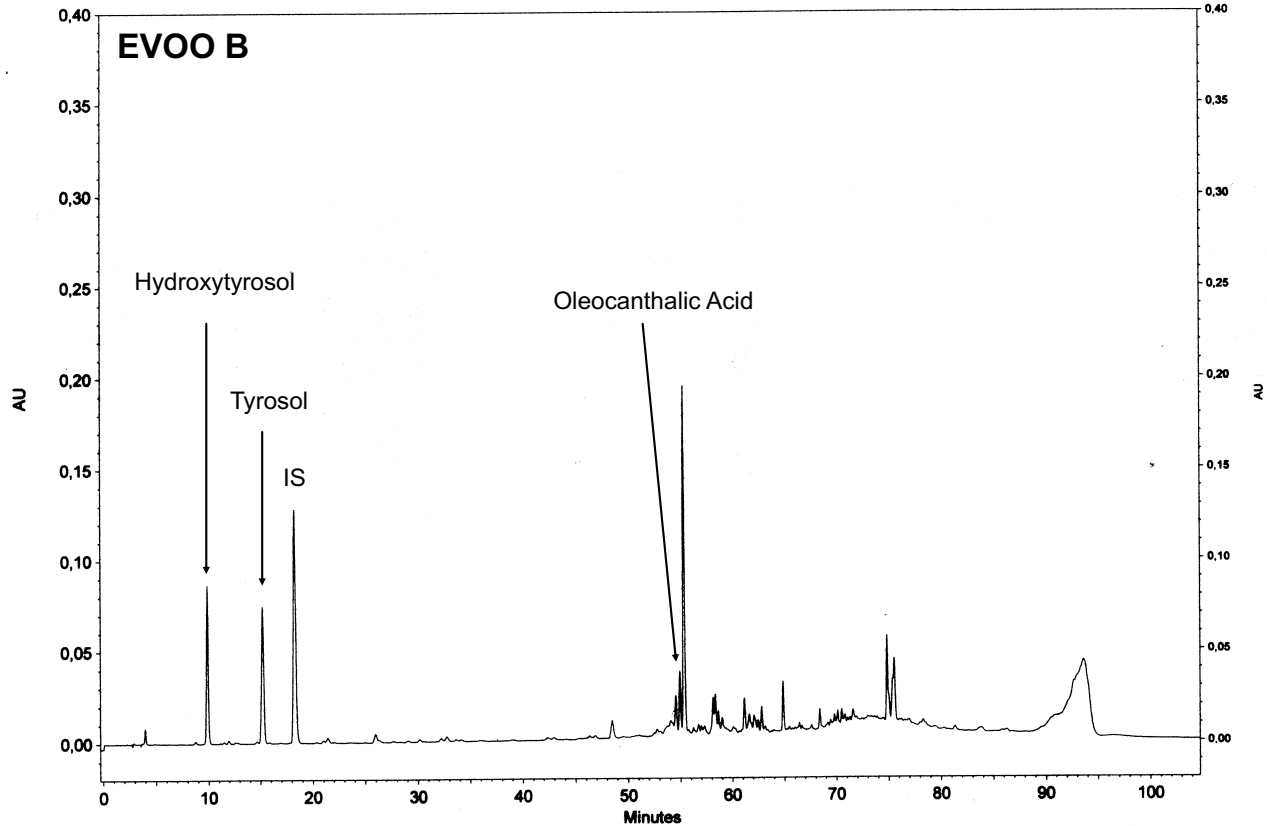

Supplement: Supplementary file 1 [file foods-11-01354-s001.zip › Figure S5.pdf]

# EVOO C

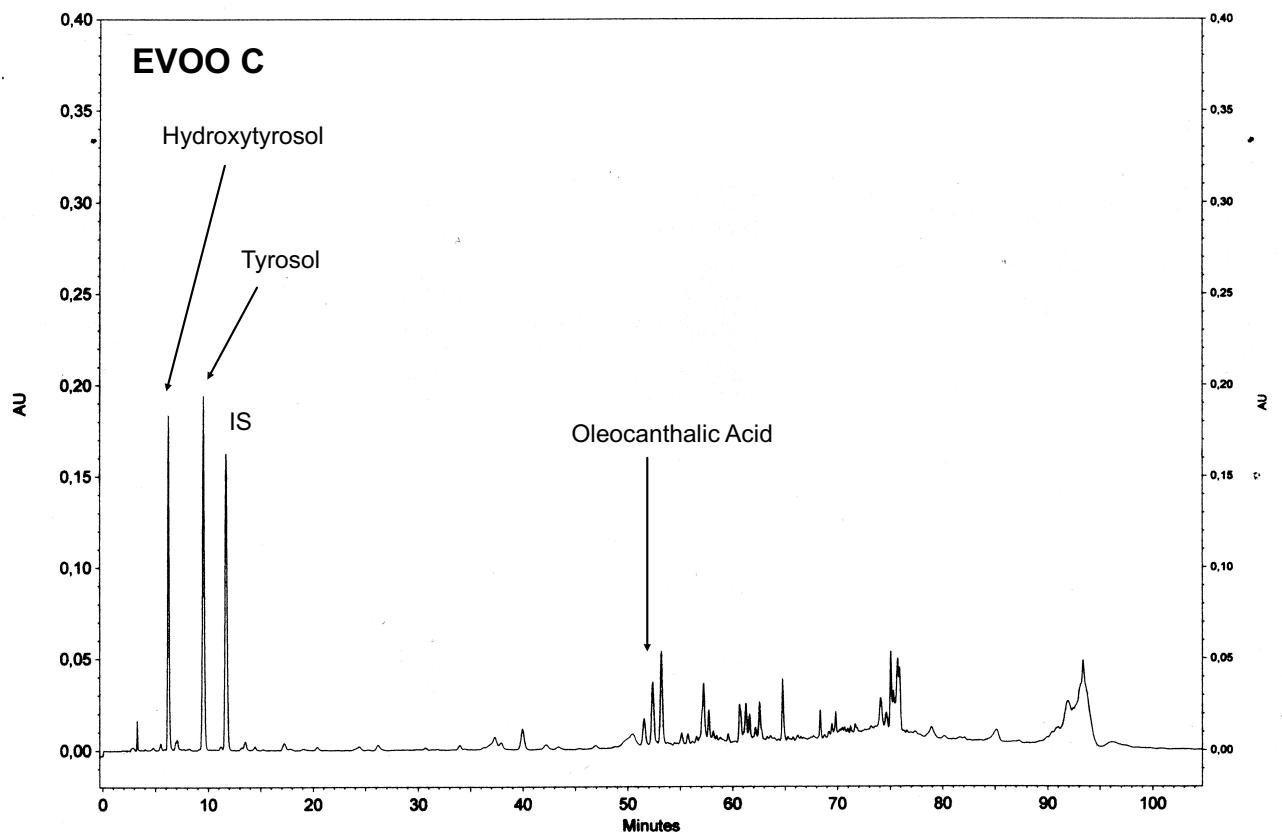

Supplement: Supplementary file 1 [file foods-11-01354-s001.zip › Figure S6.pdf]

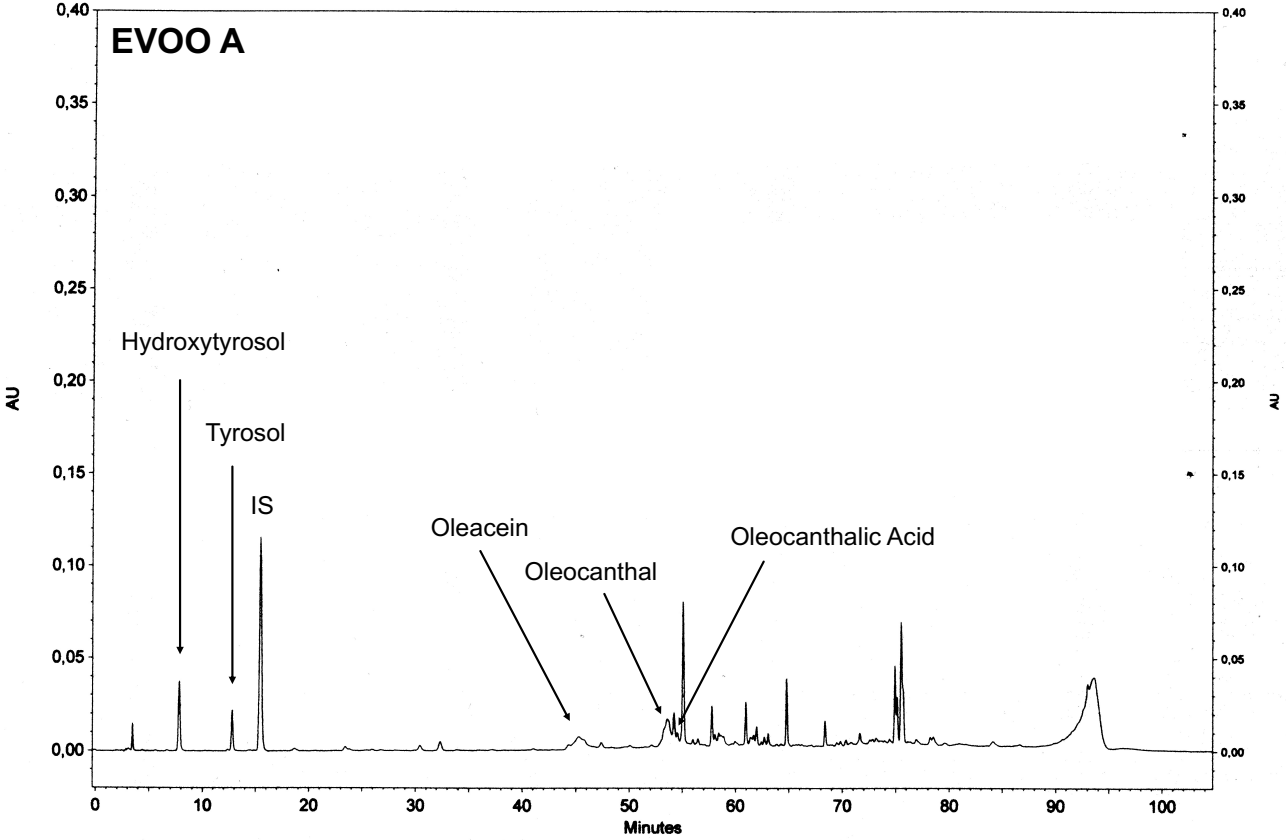

Supplement: Supplementary file 1 [file foods-11-01354-s001.zip › Figure S7.pdf]

# EVOO B

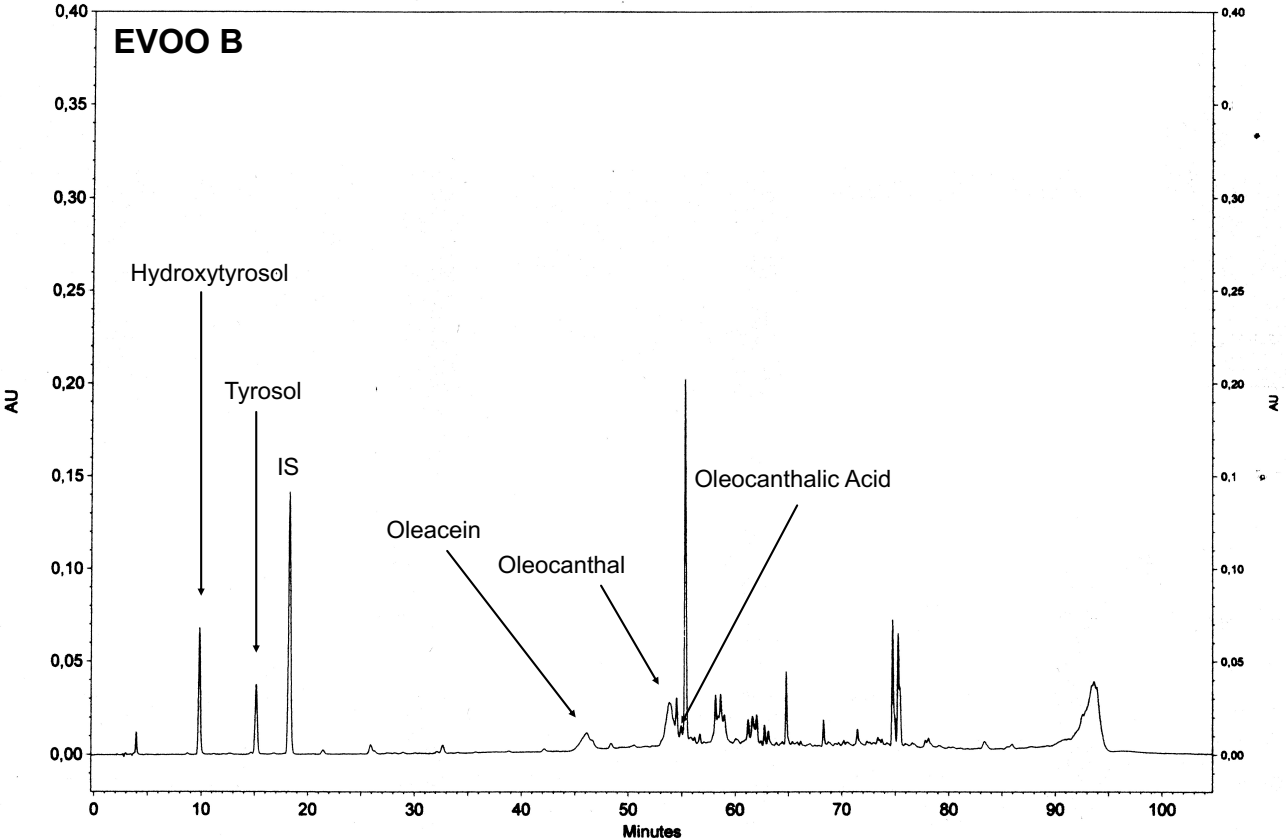

Supplement: Supplementary file 1 [file foods-11-01354-s001.zip › Figure S8.pdf]

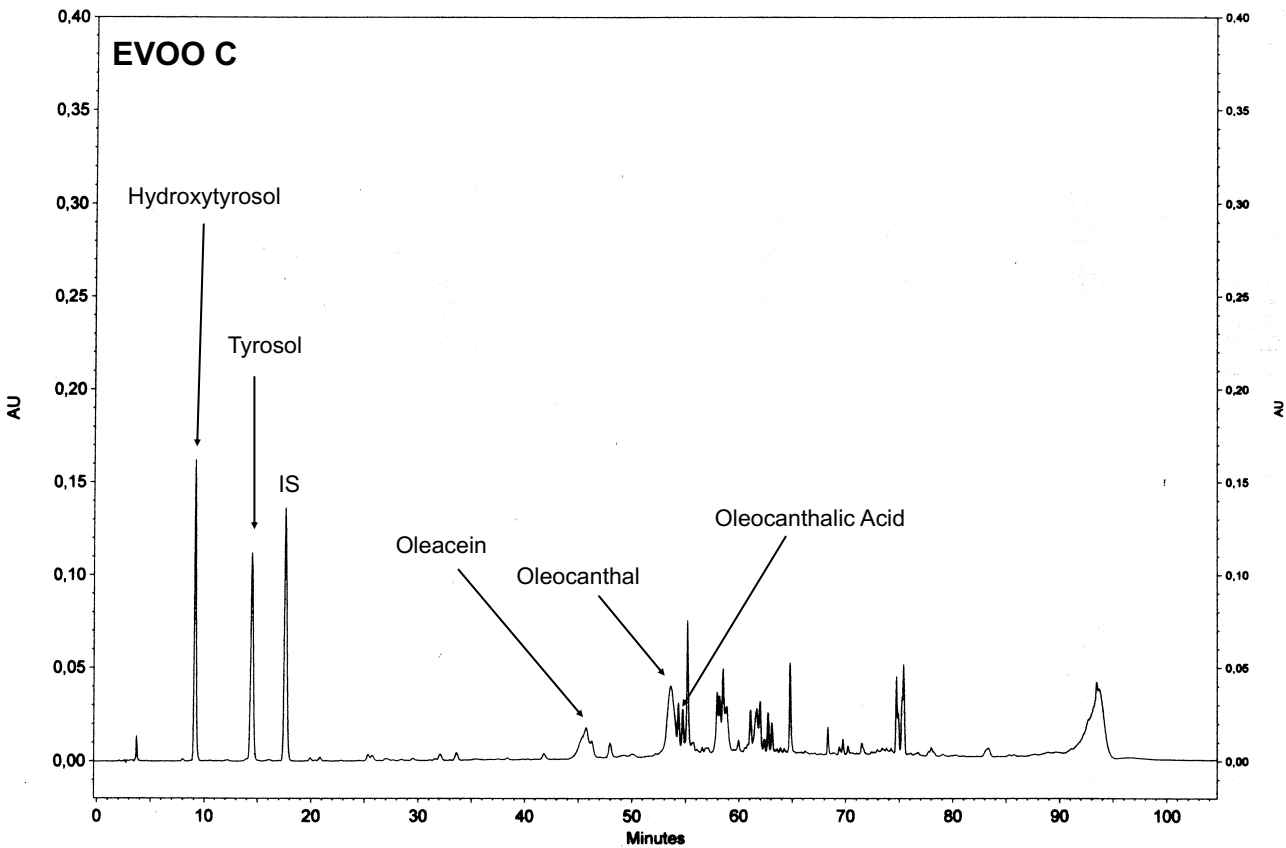

Supplement: Supplementary file 1 [file foods-11-01354-s001.zip › Figure S9.pdf]
